# The I405V and Taq1B polymorphisms of the CETP gene differentially affect sub-clinical carotid atherosclerosis

**DOI:** 10.1186/1476-511X-11-130

**Published:** 2012-10-05

**Authors:** Eliane Soler Parra, Natália Baratella Panzoldo, Denise Kaplan, Helena Coutinho Franco de Oliveira, José Ernesto dos Santos, Luiz Sérgio Fernandes de Carvalho, Andrei Carvalho Sposito, Magnus Gidlund, Ruy Tsutomu Nakamura, Vanessa Helena de Souza Zago, Edna Regina Nakandakare, Eder Carlos Rocha Quintão, Eliana Cotta de Faria

**Affiliations:** 1 Lipids Laboratory, Faculty of Medical Sciences, State University of Campinas, Rua 5 de Junho, 350, Campinas, SP 13083-877, Brazil; 2 Lipids Laboratory, Biology Institute, State University of Campinas, Rua Monteiro Lobato, 255, Campinas, SP, 13083-862, Brazil; 3 Faculty of Medicine, University of São Paulo, Av Bandeirantes, 3900, Ribeirão Preto, SP, 14039-900, Brazil; 4 Cardiology Division, Faculty of Medical Sciences, State University of Campinas, Rua Tessália Vieria de Camargo, 126, Campinas, SP, 13083-887, Brazil; 5 Imunology Department, University of São Paulo, Av Prof. Lineu Prestes, 2415, São Paulo, SP, 05508-900, Brazil; 6 Department of Radiology, Faculty of Medical Sciences, State University of Campinas, Rua Tessália Vieira de Camargo, 126, Campinas, SP, 13083-887, Brazil; 7 Lipids Laboratory (LIM 10), Faculty of Medical Sciences of the University of São Paulo, Av Dr Arnaldo, 455, São Paulo, SP, 01246-903, Brazil; 8 Departamento de Patologia Clínica, FCM-UNICAMP, P.O. Box 6111, Rua Tessália Vieira de Camargo, 126, Campinas, Barão Geraldo, SP 13084-971, Brazil

**Keywords:** Carotid atherosclerosis, Carotid intima-media thickness, Cholesteryl ester transfer protein, Genetic polymorphism

## Abstract

**Background:**

Cholesteryl ester transfer protein (CETP) plays a major role in lipid metabolism, but studies on the association of CETP polymorphisms with risks of cardiovascular disease are inconsistent. This study investigated whether the CETP gene I405V and Taq1B polymorphisms modified subclinical atherosclerosis in an asymptomatic Brazilian population sample.

**Methods:**

The polymorphisms were analyzed using polymerase chain reaction in 207 adult volunteers. Serum lipid profiles, oxLDL Ab titers, C-reactive protein and tumor necrosis factor-α concentrations and CETP and phospholipid transfer protein (PLTP) activities were determined, and common carotid artery intima-media thickness (cIMT) was measured using ultrasonography.

**Results:**

No differences in cIMT were observed between the presence or absence of the minor B2 and V alleles in either polymorphism. However, inverse correlations between mean cIMT and CETP activity in the presence of these polymorphisms were observed, and positive correlations of these polymorphisms with PLTP activity and oxLDL Ab titers were identified. Moreover, logistic multivariate analysis revealed that the presence of the B2 allele was associated with a 5.1-fold (CI 95%, OR: 1.26 – 21.06) increased risk for cIMT, which was equal and above the 66^th^ percentile and positively interacted with age. However, no associations with the V allele or CETP and PLTP activities were observed.

**Conclusions:**

None of the studied parameters, including CETP activity, explained the different relationships between these polymorphisms and cIMT, suggesting that other non-determined factors were affected by the genotypes and related to carotid atherosclerotic disease.

## Background

Atherosclerotic disease is a complex multifactorial process that leads to the deposition and accumulation of cholesterol along the arterial wall [1]. Cholesteryl ester transfer protein (CETP) plays a major role in cholesterol metabolism [2], and polymorphisms within this gene may underlie the susceptibility to atherosclerosis [3].

The I405V (rs5882) and Taq1B (rs708272) polymorphisms and their respective minor alleles, V and B2, are associated with decreased CETP activity and increased high-density lipoprotein cholesterol (HDL-C) levels [4]. However, studies on the association of these polymorphisms with risks of cardiovascular disease (CVD) are inconsistent [5], and their association with subclinical CVD are less well characterized.

Therefore, this study investigated whether I405V and Taq1B polymorphisms modified subclinical atherosclerosis in an asymptomatic Brazilian adult sample population.

## Methods

### Study subjects

A total of 207 asymptomatic volunteers of both sexes (20–75 years) were recruited at the Lipid Outpatient Clinic at the State University (Unicamp) Hospital in Campinas, SP, Brazil.

The following exclusion criteria were used: body mass index (BMI) ≥ 30 kg/m^2^; a diagnosis of endocrinological, liver or kidney disease; use of drugs that alter the lipid profile; excessive use of alcohol (>75 g/day) [6]; meeting the “present CVD” criteria (acute myocardial infarction and angina pectoris, as established by electrocardiogram, cardiac-specific enzyme levels, coronary angiography and/or a history of coronary angioplasty or myocardial revascularization).

The local Medical School Ethics Committee approved this study, and all participants signed an informed consent form.

#### Biochemical analysis

The following blood measurements were performed using Roche Diagnostics (Mannheim, Germany) reagents in an automated system: total cholesterol (CHOD-PAP), triglycerides (TG, GPO-PAP), HDL-C (HDL-C plus 3rd generation), low-density lipoprotein cholesterol (LDL-C plus 2nd generation for TG >400 mg/dL and Friedewald’s equation for TG ≤ 400 mg/dL). Apolipoproteins AI (NAS-ApoA1), B100 (NAS-ApoB) and lipoprotein (a) were obtained from Siemens (USA). C-reactive protein (CRP) was measured using the Tina-quant® CRP (latex) high sensitivity assay (Roche Diagnostics, Switzerland). Plasma levels of tumor necrosis factor α (TNF-α) were measured using ELISA (Cayman Chemical, USA).

HDL_2_ and HDL_3_ sub-fractions were obtained using sequential micro-ultracentrifugation [7].

CETP [8] and phospholipid transfer protein (PLTP) activities [9] in plasma were determined using radioassays with exogenous substrates.

Antibodies against oxidized LDL (oxLDL Ab) titers were determined using an in-house ELISA [10].

### Polymorphism detection

Genomic DNA was extracted [11] and amplified using polymerase chain reaction to identify the Taq1B [12] and I405V [13] polymorphisms.

### Measurement of cIMT

A single trained sonographer, who was blind to the subject’s identity, performed high-resolution B-mode common carotid ultrasonography using a 6–9 MHz linear array ultrasound imaging system (ATL HDI 1500 Ultrasound System, Advanced Technology Laboratories Ultrasound, USA). Longitudinal measurements of segments of the common carotid arteries at the distal wall and 1 cm from the bifurcation were performed according to a standardized method [14]. The mean common carotid artery intima-media thickness (cIMT) was calculated as the average of five measurements on each side (right and left) and expressed in millimeters.

### Statistical analysis

Mann–Whitney U and Chi-Squared tests compared variables, and the Spearman test was performed for correlations. Univariate and multivariate logistic regression analyses, with stepwise criteria of the variables of interest, the presence of the V and B2 allele, gender, age, HDL-C, BMI and systolic blood pressure (SBP), were performed to verify the relationships of these variables with higher cIMT values, which was defined as above or below the established cutoff value of 0.795 mm and corresponding to cIMT equal and above the 66^th^ percentile values of the studied population.

All the analyses were performed using the SAS®, version 9.1.3 and SPSS 11.5 for Windows. Haploview, v 4.2^©^ was used for the linkage disequilibrium (LD) analysis [15].

## Results

The frequencies of I405V and Taq1B genotypes in our population sample, represented by the presence (VV, IV) and absence (II) of the V allele and the presence (B2B2, B1B2) and absence (B1B1) of the B2 allele, are presented in Table 1. The frequencies of the II, IV and VV genotypes were 28%, 53% and 19%, respectively, and the frequencies of the B1B1, B1B2 and B2B2 were 33%, 41% and 26%, respectively.

**Table 1:** Biochemical parameters in the presence and absence of minor alleles

**Parameters**	**A. I405V Genotypes**		
	**II**	**V carriers**	**p value ≤**
	**(n=32-59)**	**(n=70-148)**	
**Frequency (%)**	**29**	**71**	
**CETP (%)**	**14.5 ± 9.0**	**11.0 ± 7.2**	**0.015**
HDL-C (mg/dL)	72 ± 24	74 ± 18	0.192
**HDL**_**2**_**-C (mg/dL)**	**15 ± 6**	**17 ± 6**	**0.030**
**PLTP (%)**	**22.3 ± 11.9**	**15.8 ± 9.9**	**0.001**
Lipoprotein (a) (mg/dL)	27 ± 30	32 ± 36	0.477
OxLDL Ab (OD)	0.33 ± 0.16	0.38 ± 0.19	0.405
**Parameters**	**B. Taq1B Genotypes**		
	**B1B1**	**B2 carriers**	**p value ≤**
	**(n=32-68)**	**(n=70-139)**	
**Frequency (%)**	**33**	**67**	
CETP (%)	13.4 ± 8.2	11.3 ± 7.7	0.103
HDL-C (mg/dL)	74 ± 26	73 ± 16	0.584
HDL_2_-C (mg/dL)	17 ± 6	17 ± 5	0.657
PLTP (%)	17 ± 11	18 ± 11	0.860
**Lipoprotein (a) (mg/dL)**	**24 ± 30**	**33 ± 36**	**0.014**
oxLDL Ab (OD)	0.33 ± 0.17	0.38 ± 0.18	0.230

Data are presented as means ± standard deviation; (sample number): n = minimum and maximum of subjects with determined parameters; p values: Mann–Whitney *U* test for comparisons between the presence and absence of the minor alleles (V and B2). Significant differences: p≤0.05 in bold. CETP (%) = activity of cholesterol ester transfer protein; HDL-C = high-density lipoprotein cholesterol; PLTP (%) = activity of phospholipid transfer protein; oxLDL Ab = antibodies against oxidized LDL titers; (OD)= optical density.

Allele frequencies were consistent with Hardy–Weinberg equilibrium for the I405V polymorphism (p=0.36) but not the Taq1B (p=0.02), and the MAFs (minor allele frequency) were <45% and <46%, respectively. The linkage disequilibrium between the two polymorphisms was very weak, as evidenced by the D`=0.17.

Clinical and anthropometric characteristics and biochemical data of the investigated population are presented in Tables 2 and 1.

**Table 2:** Clinical and anthropometric characteristics in the presence and absence of minor alleles

**Parameters**	**A. I405V Genotypes**		
	**II**	**V carriers**	**p value ≤**
	**(n=34-59)**	**(n=83-148)**	
**Age (years)**	47 ± 14	49 ± 15	NS
**Male (%)**	24	26	NS
**BMI (kg/m**^**2**^**)**	25 ± 6	25 ± 5	NS
**WC (cm)**	**76 ± 11**	**81 ± 12**	**0.008**
**SBP (mmHg)**	**120 ± 13**	**127 ± 16**	**0.002**
**DBP (mmHg)**	**77 ± 10**	**82 ± 10**	**0.001**
**Mean cIMT (mm)**	0.74 ± 0.19	0.79 ± 0.23	NS
**Parameters**	**B. Taq1B genotypes**		
	**B1B1**	**B2 carriers**	**p value ≤**
	**(n=37-68)**	**(n=80-139)**	
**Age (years)**	49 ± 15	48 ± 14	NS
**Male (%)**	**35**	**21**	**0.026**
**BMI (kg/m**^**2**^**)**	25 ± 4	25 ± 6	NS
**WC (cm)**	81 ± 12	79 ± 12	NS
**SBP (mmHg)**	127 ± 14	124 ± 16	NS
**DBP (mmHg)**	82 ± 11	79 ± 10	NS
**Mean cIMT (mm)**	0.73 ± 0.20	0.79 ± 0.23	NS

Data presented as means ± standard deviation; (sample number): n = minimum and maximum of subjects with determined parameters; p values = Mann–Whitney *U* test for comparisons between the presence and absence of the alleles (V and B2); gender comparisons, Chi-Square test. Significant differences: p≤0.05 in bold. BMI= Body Mass Index; WC= waist circumference; SBP= systolic blood pressure; DBP= diastolic blood pressure; cIMT= carotid intima-media thickness.

Table 3 presents the significant correlations between mean cIMT, oxLDL Ab titers and CETP and PLTP activities.

**Table 3:** Significant correlations between cIMT, oxLDL Ab, CETP (%) and PLTP (%) in the presence of minor alleles

**cIMT *versus***	**V carriers**		**cIMT *versus***	**B2 carriers**	
	**r**	**p value ≤**		**r**	**p value ≤**
**CETP (%) n=88**	**−0.363**	**0.0001**	**CETP (%) n=87**	**−0.427**	**0.0001**
**oxLDL Ab (OD) n=54**	**0.409**	**0.002**	**oxLDL Ab (OD) n=51**	**0.432**	**0.002**
**PLTP (%) n=85**	**0.322**	**0.003**	**PLTP (%) n=84**	**0.315**	**0.004**

r= Spearman´s coefficient; Significant differences: p≤0.05 in bold. cIMT= carotid intima-media thickness; oxLDL Ab= antibodies against oxidized LDL titers; (OD) = optical density; CETP(%) = activity of cholesteryl ester transfer protein; PLTP (%) = activity of phospholipid transfer protein.

The univariate analyses (Table 4) indicated that the female gender, age, HDL-C, BMI and SBP were significantly associated (*p* value ≤0.05) with higher cIMT.

**Table 4:** **Univariate Logistic Regression analyses for higher cIMT (above 66 percentile) (*n=*118)**

**Variables**	**Categories**	**p value ≤**	**OR**	**CI 95% OR**
Presence of V allele	No (ref).	0.303	1.00	—
	Yes		1.57	0.67 – 3.69
Presence of B2 allele	No (ref).	0.150	1.00	—
	Yes		1.87	0.80 – 4.36
**Gender**	Male (ref.)		1.00	—
	**Female**	**0.048**	**2.48**	**1.01 – 6.10**
**Age (years)**	**(cont).**	**0.001**	**1.195**	**1.116 – 1.279**
**HDL-C (mg/dL)**	**(cont).**	**0.035**	**1.024**	**1.002 – 1.047**
**BMI (kg/m**^**2**^**)**	**(cont).**	**0.009**	**1.105**	**1.025 – 1.191**
**SBP (mmHg)**	**(cont).**	**0.003**	**1.043**	**1.014 – 1.072**

cIMT below and above the 66^th^ percentile, <0.795 (n=76) and >0.795 (n=42), respectively. (ref).=reference level. (cont).= continue variable. OR= Odds ratio for higher cIMT, above the 66^th^ percentile value (0.795 mm), CI 95% OR= Confidence interval of 95% for the Odds ratio. Significant differences: p≤0.05 in bold; B2 = minor allele of Taq1B polymorphism, V = minor allele of I405V polymorphism, HDL-C = high-density lipoprotein cholesterol, BMI: body mass index, SBP: systolic blood pressure.

The multivariate analysis (Table 5) demonstrated that age and the presence of the B2 allele were positively related to higher cIMT. Individuals with increased risk exhibited the B2 allele (5.1-fold increase) and higher age (21.2% increase/each year of age).

**Table 5:** Multivariate Logistic Regression* analyses for higher cIMT (above 66^**th**^**percentile) (*n=*114)**

**Variables**	**Categories**	**p value ≤**	**OR**	**CI 95% OR**
**Age (years)**	**(cont).**	**0.001**	**1.212**	**1.126 – 1.304**
**Presence of B2 allele**	No (ref).	**0.023**	1.00	**—**
	**Yes**		**5.14**	**1.26 – 21.06**

*Stepwise criteria for selection of variables. cIMT below and above 66^th^ percentile, <0.795 (n=73) and >0.795 (n=41), respectively. (ref).= reference level; (cont).=continue variable. OR= Odds ratio for higher cIMT above the 66^th^ percentile value (0.795 mm), CI 95% OR= Confidence interval of 95% for Odds ratio. Significant differences: p≤0.05 in bold; B2 = minor allele of Taq1B polymorphism.

A Chi-Square analysis indicated an interaction between the presence of the B2 allele and age. The combination between age, categorized in ≥ and <50 years (median value), and the presence or absence B2 allele formed 4 groups of individuals who were crossed with cIMTs above and below 0.795 mm (data not shown).

## Discussion

This study investigated whether the I405V and Taq1B polymorphisms modify subclinical atherosclerosis in an asymptomatic population sample.

Previous studies present controversial data on the role of Taq1B and I405V in atherosclerosis and CVD. The B2 allele is associated with lower internal carotid artery IMT in men [16], but other studies do not identify associations between the Taq1B polymorphism and cIMT [17,18]. The present study demonstrated that cIMT did not differ between either allele of the polymorphisms. However, inverse correlations between cIMT and CETP activity were demonstrated in both polymorphisms (Table 3), which suggest a potential atheroprotective role for CETP in these individuals. A potential anti-inflammatory role for CETP was demonstrated previously [19] wherein the induction of acute inflammation produced lower mortality due to sepsis and lower cytokine circulation in mice that express human CETP. Reductions in CETP concentrations in human patients with sepsis are more pronounced in non-survivors [20].

We identified positive correlations between cIMT and PLTP and oxLDL Ab in the presence of the V and B2 alleles. Previous studies have suggested that PLTP increases the risk of cardiovascular disease in humans [21], and the balance of evidence suggests a pro-atherogenic role for oxLDL Ab titers [22] in the development of atherosclerosis. These positive correlations and the lack of correlations of these variables in both common B1B1 and II allele homozygotes (CETP p≤0.318 and 0.091, oxLDL Ab p≤ 0.709 and 0.386 and PLTP p≤0.823 and 0.470, respectively) (data not shown) suggest a possible pro-atherogenic role of these markers associated with the polymorphisms. Our sample size was relatively small and different between groups. Therefore, further studies are underway in our lab to further confirm these correlations.

The presence of the V allele was associated with lower CETP (24%) and PLTP (29%) activities and higher HDL_2_-C (13%) concentrations. Patients with coronary disease typically exhibit fewer large HDL particles (HDL_2_), and the concentration of these large particles is a better predictor of future cardiovascular events than HDL-C concentration [23].

Lipoprotein (a) plasma levels were also significantly increased in the presence of the B2 allele. High levels of lipoprotein (a) are significantly associated with the presence, severity and extent of carotid atherosclerosis [24], but this increase was not observed as a possible pro-atherogenic factor.

No significant differences in CETP activity and HDL-C levels were observed in Taq1B polymorphism, despite a 15.4% reduction in CETP activity in presence of the B2 allele. These results are consistent with a previous study in another Brazilian population, in which no association between Taq1B genotypes and HDL-C levels was observed [25].

A univariate analysis (Table 4) followed by a multivariate logistic stepwise analysis (Table 5) demonstrated that the presence of the B2 allele was independently associated with a mean 5.1-fold increased risk for higher cIMT and age interacted with the allele effect. The presence of the B2 allele in the ≥ 50 years age group exhibited the highest risk of higher cIMT in the Chi-Square analysis (data not shown).

The effects of the Taq1B variant on circulating plasma lipids may be related to other functional mutations within the CETP gene locus that are in LD [26], which may explain the association in our study. However, other factors than the CETP activity may exert different atherosclerotic repercussions in Taq1B and I405V polymorphisms. A multivariate analysis was performed to verify the independence of the associations between the qualitative binary variable cIMT above or below 66^th^ percentile (0.795mm), CETP and PLTP plasma activities and the potential confounding factors, including gender, age, BMI, SBP and HDL-C. Only age exhibited a significant statistical association (OR: 1.247, CI 95%OR: 1.129 – 1.377), which was a strong determinant of cIMT (data not shown). The absence of interactions of other tested parameters with higher cIMT in this study suggests that other non-determined factors were affected by the genotypes and related to carotid atherosclerotic disease.

Our results focused on several biomarkers that were assessed for the first time in the Brazilian population, and the results were partially based on the correlations between a relatively small numbers of individuals. These results merit further exploration due to the high frequency of the investigated polymorphisms in the present Brazilian sample. Identifying the impact of these polymorphisms is an ongoing aim of our research group.

## Conclusions

The I405V and Taq1B polymorphisms were differently associated with subclinical atherosclerosis because only the presence of the minor B2 allele, but not the V allele, was independently associated with higher cIMT. We demonstrated that none of the studied parameters explained the different relationships between these polymorphisms and cIMT, which leads us to speculate that the association between the B2 allele and cIMT is due to other non-studied factors. Ongoing studies are underway in our laboratory to determine the modulation factors that are involved.

## Abbreviations

CETP: Cholesteryl Ester Transfer Protein; HDL-C: High-Density Lipoproteins Cholesterol; CVD: Cardiovascular Disease; BMI: Body Mass Index; TG: TriGlycerides; LDL-C: Low-Density Lipoprotein Cholesterol; Apo: Apolipoprotein; CRP: C-Reactive Protein; TNF-α: Tumor Necrosis Factor-α; PLTP: Phospholipid Transfer Protein; oxLDL Ab: Antibodies titers against oxidized LDL; cIMT: common Carotid Artery Intima-Media Thickness; SBP: Systolic Blood Pressure; LD: Linkage Disequilibrium; MAF: Minor Allele Frequency.

## Misc

Eliane Soler Parra and Natália Baratella Panzoldo contributed equally to this manuscript.

## Competing interests

The authors state that they have no conflicts of interest.

## Authors’ contributions

ESP worked hard on the data analysis, manuscript preparation, and editing. NBP wrote and reviewed the final manuscript. DK participated in the selection of participants, collection of all clinical and laboratory data and clinical examinations. HCFO and JES assisted in the execution of the experimental protocol for the polymorphism detection. LSFC and ACS performed additional statistical analyses and critical discussion. MG conducted the immunological experiments. RTN performed the carotid ultrasonography. VHSZ, ERN and ECRQ critically revised all text and complemented the discussion. ECF designed the study, implemented the clinical arm and coordinated the work. All authors read an approved the manuscript.
